# Designing Artificial Intelligence-Powered Health Care Assistants to Reach Vulnerable Populations: A Discrete Choice Experiment Among South African University Students

**DOI:** 10.1016/j.mcpdig.2025.100248

**Published:** 2025-07-10

**Authors:** Amy Zheng, Lawrence Long, Caroline Govathson, Candice Chetty-Makkan, Sarah Morris, Dino Rech, Matthew P. Fox, Sophie Pascoe

**Affiliations:** a Department of Epidemiology, Boston University School of Public Health, Boston, MA; b Department of Global Health, Boston University School of Public Health, Boston, MA; c Health Economics and Epidemiology Research Office, Faculty of Health Sciences, University of the Witwatersrand, Johannesburg, South Africa; d Audere, Seattle, WA

## Abstract

**Objective:**

To understand what preferences are important to university students in South Africa when engaging with a hypothetical artificial intelligence-powered health care assistant (AIPHA) to access health information using a discrete choice experiment.

**Patients and Methods:**

We conducted an unlabeled, forced choice discrete choice experiment among adult South African university students through Prolific, an online research platform, from June 26, 2024 to August 31, 2024. Each choice option described a hypothetical AIPHA using 8 attribute characteristics (cost, confidentiality, security, health care topics, language, persona, access, and services). Participants were presented with 10 choice sets each comprised of 2 choice options and asked to choose between the 2. A conditional logit model was used.

**Results:**

Three hundred participants were recruited and enrolled. Most participants were Black, born in South Africa, heterosexual, working for a wage, and had a mean age of 26.5 years (SD, 6.0). Language, security, and receiving personally tailored advice were the most important attributes for AIPHA. Participants strongly preferred the ability to communicate with the AIPHA in any South African language of their choosing instead of only English and receive information about health topics specific to their context including information on clinics geographically near them. The results were consistent when stratified by sex and socioeconomic status.

**Conclusion:**

Participants had strong preferences for security and language, which is in line with previous studies where successful uptake and implementation of such health interventions clearly addressed these concerns. These results build the evidence base for how we might engage young adults in health care through technology effectively.

Accessing appropriate sexual and reproductive health (SRH) services for adolescents and young adults is critical to prevent unintended pregnancies, the transmission of sexually transmitted infections (STIs), and human immunodeficiency virus (HIV). A key component of being able to access SRH services is knowledge and education. In South Africa, where HIV is one of the leading causes of morbidity and mortality, low SRH knowledge among young adults, including university students, contributes to high-risk behaviors, and in turn increases their risk of unintended pregnancies, transmission of STIs, and HIV.^1^, ^2^, ^3^, ^4^, ^5^, ^6^ This limited knowledge may help explain, in part, the underrepresentation of this group in HIV prevention services, despite being disproportionately affected by HIV.^1^^,^^4^^,^^7^, ^8^, ^9^, ^10^, ^11^ University students may be at increased risk due to recent transitions away from home and exposure to new social and sexual environments.^6^^,^^7^^,^^12^^,^^13^ Although attendance at university presents a unique opportunity to provide a greater degree of SRH services due to the physical concentration of students on campus, SRH services, in particular HIV care and prevention, are often limited particularly at under-resourced institutions.^6^^,^^12^, ^13^, ^14^ Furthermore, South African university students have a high prevalence of mental health conditions such as anxiety, depression, and substance use, which may impact the willingness and ability to access in-person health services.^15^, ^16^, ^17^ Being able to reach young adult South Africans, provide them with appropriate health information, identify their health care needs, and provide appropriate health services remains a critical gap in improving their overall health and reaching HIV epidemic control.

Young adults experience substantial barriers in accessing health care services including low health literacy, long wait times at health facilities, academic obligations, and negative experiences with health care workers.^14^^,^^15^^,^^18^, ^19^, ^20^, ^21^ Long wait times may be a stronger deterrent for young adults who are not ill and are only seeking information and preventive services. Similarly, health care worker attitude may be particularly important for those seeking SRH services that are often stigmatized. Recent advances in artificial intelligence (AI), in particular AI-powered health care assistants (AIPHAs) built on large-language models (LLMs), present an opportunity to address some of these barriers and help countries achieve the United Nations Sustainable Development Goal 3.7, ensuring universal access to SRH information, education, and services.^22^, ^23^, ^24^, ^25^ Within the health sector, AIPHAs have been used for tasks ranging from providing health information and risk screening to scheduling appointments and providing diagnostic support.^23^^,^^24^^,^^26^, ^27^, ^28^, ^29^, ^30^, ^31^ Preliminary work suggests that AIPHAs could be particularly suited to educating clients and facilitating referrals for topics that are potentially stigmatizing, like sexual history and HIV status. However, their success depends on tailoring the design and delivery to meet the needs and preferences of the end users.

Despite the exponential growth of AIPHAs, there is a dearth of data about which characteristics end users value and would drive uptake.^32^^,^^33^ As most South Africans have to pay for internet data, cost has been found to affect South African health care users’ decisions and thus may be important for AIPHAs.^34^^,^^35^ Similarly, if we draw parallels from the concerns clients have raised with in-person health interactions, characteristics such as confidentiality (ie, password protection and storage of data), the interaction experience (ie, friendliness and personality of the tool persona), and the breadth of knowledge (ie, conditions covered and knowledge of relevant services) may be important for decision-making.^32^^,^^33^^,^^36^ Structural questions, such as how the health application is accessed (ie, online, downloaded application) and in what language, are likely to influence use in places like South Africa where most of the population only has access to basic smartphone capabilities and there is language diversity (11 official spoken South African languages).^34^ Although individual characteristics have been explored in prior studies, to our knowledge, no evaluation of the importance of these characteristics alongside 1 another in decision-making supported by AIPHAs has been conducted.^23^^,^^24^^,^^29^^,^^31^^,^^37^, ^38^, ^39^

Given the growing use of AIPHAs and their potential to address gaps in the HIV care cascade in resource-limited health care settings, we sought to identify which attributes (or characteristics) users value in deciding whether to use an AIPHA. Recognizing that preferences vary by context and user group, we enrolled tertiary students in South Africa.^39^ We conducted a discrete choice experiment (DCE) to determine South African tertiary students’ preferences for a specific attribute when decided across various AIPHAs.^40^

## Patients and Methods

### Advisory Committee

An 8-person advisory committee provided technical oversight and local context when considering the selection of attributes (the characteristics of a choice option) to include in the DCE. This committee included the research implementation team; South African researchers with experience in DCEs, HIV prevention, and working with vulnerable populations; and AI technology experts with implementation experience in South Africa. The advisory committee was involved in all major decisions regarding study design, analysis, and interpretation and provided guidance on attributes and their associated levels (the values that the characteristic can assume).^40^^,^^41^ As there were no reliable user cost estimates for similar tools in this context, we included cost as a categorical variable instead of a continuous measure.

### Development of Attributes and Levels and Ranking Survey

An initial literature search used PubMed to identify and create a provisional list of modifiable attributes of a hypothetical AIPHA (see search terms in Supplemental Table 1, available online at https://www.mcpdigitalhealth.org/).^6^^,^^13^, ^14^, ^15^^,^^20^^,^^31^^,^^38^^,^^39^^,^^42^, ^43^, ^44^ This search included articles in English from 2015 to 2023 (excluding preprints), focused on health care services likely to be important for and provided to South African university students. The advisory committee reviewed and augmented the list to create 13 overarching attributes (Supplemental Table 2, available online at https://www.mcpdigitalhealth.org/) they believed were important when developing and utilizing an AIPHA. Discrete choice experiment best practice suggests approximately 8 attributes with at most 4 levels per attribute to ensure that participants are not overburdened by the different choice options. Utilizing Prolific, an online research platform, we then conducted a survey on April 11, 2024 asking 30 adult university students (≥18 years old) in South Africa to rank these 13 attributes of an AIPHA from most to least important when engaging with an AIPHA. Use of Prolific in behavioral research such as DCEs has been well-established.^45^^,^^46^ To create a single “super” list that was reflective of all participants, we conducted a rank aggregation using the Cross-Entropy Monte Carlo algorithm (Supplemental Appendix 1, available online at https://www.mcpdigitalhealth.org/).^47^^,^^48^ Based on this ranking and input from the advisory committee, we identified the 8 attributes likely to be the most important. The final 8 attributes and the possible levels were presented to the South African research team to ensure appropriateness and clarity in language (Table 1) and were then used to design the DCE.

**Table 1:** Attributes and Levels of the Discrete Choice Experiment

Attribute	Levels
Cost	Free to use Uses Data
Confidentiality	Data are deleted immediately and cannot be used in future uses Data are stored for 30 d, and during the 30 d window, data can be used anytime the electronic tool is accessed Data are stored permanently and can be used anytime the electronic tool is accessed
Security level	No information is required to use the tool A password and either a telephone number of email is required to use the tool A password, email, and telephone number is required to use the tool
Health care topics the tool covers	HIV/STI prevention information only HIV/STI prevention and family planning information only All health conditions including HIV/STI prevention
Language	Only English English and Slang (eg, Language you would use with your friends) All official languages in South Africa
Personality of the tool	No personality A trusted friend A health care worker
How you will access the tool	Application that needs to be downloaded Uses an existing application (Ex. WhatsApp) Internet website
Type of advice the tool provides	Explain medical conditions/procedures and likely course of treatment Explain medical conditions/procedures, likely course of treatment, and advice specific to you Explain medical conditions/procedures, likely course of treatment, advice specific to you, and recommend clinics near you

### Survey Instrument Design and Implementation

**Figure 1:**
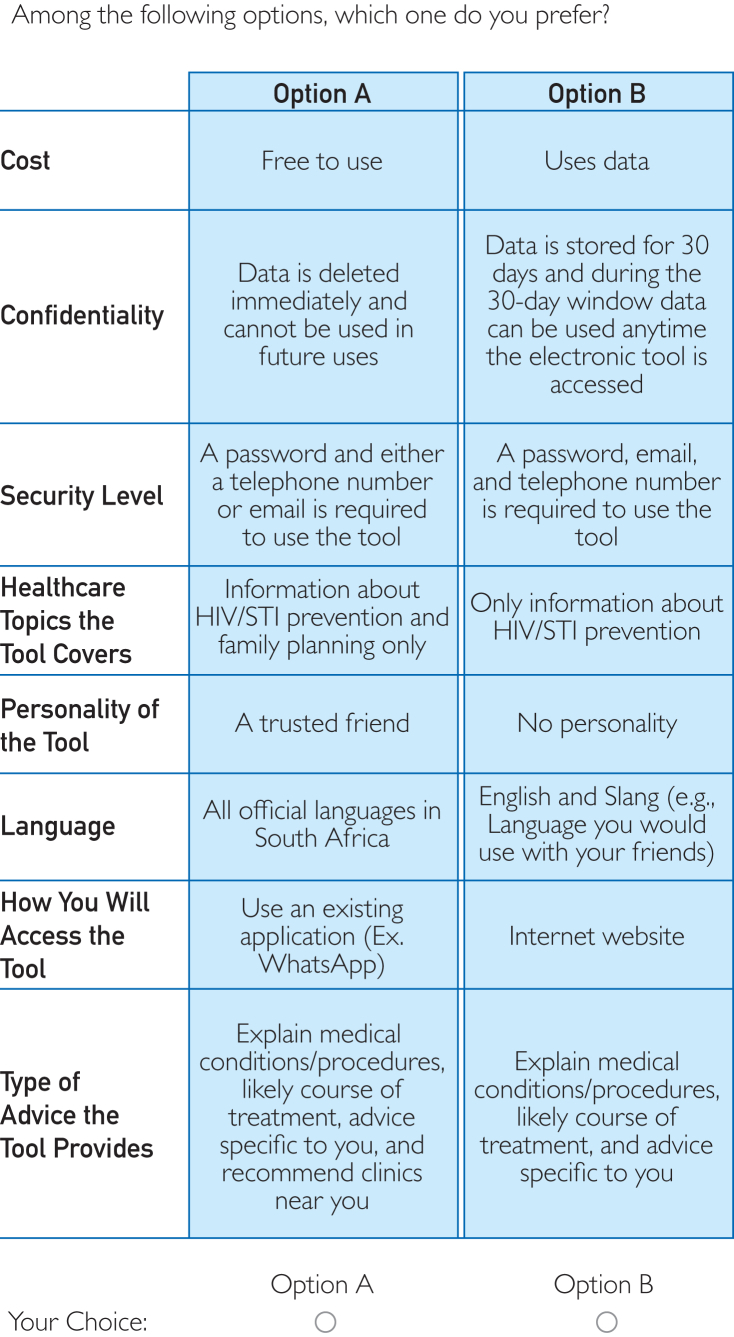
Example choice set that is presented to the participants.

The survey instrument consisted of sociodemographic questions and DCE choice sets (10 choice sets per participant). Sociodemographic questions were adapted from standardized questions used in South Africa and included questions on age, sex, sexual orientation, province of residence, socioeconomic status, and medical insurance status. We conducted an unlabeled, forced choice DCE asking participants to choose between 2 alternatives (A and B), where each alternative had 8 attributes set at different levels. Figure 1 presents an example choice set with 2 alternatives.

We used Stata to generate a *D-efficient* experimental design to maximize the information contained in each choice set using the modified Fedorov algorithm.^49^ Supplemental Tables 3 and 4 (available online at https://www.mcpdigitalhealth.org/) demonstrate that the choice sets were balanced and orthogonal. The experimental design had 18 choice sets from which we created 2 blocks. To prevent respondent fatigue and cognitive overload, each participant saw only 9 unique choice sets.^40^^,^^50^ We included a duplicate choice set to measure internal consistency, each participant therefore completed 10 choice sets. Participants were randomized 1:1 to each block. The survey was administered in English and implemented using Qualtrics, an online survey platform.^51^ Prior to completing the DCE participants watched a brief video explaining the attributes, levels, and data collection activities for the DCE.

### Sample Size Estimation

The Johnson and Orme formula was used to determine the appropriate sample size (Equation 1) due to the lack of prior preference estimates.^52^ Based on this formula, with 10 choice sets and 4 levels, a minimum sample size of 100 was required. This was then increased to 150 based on literature that suggests substantial increases in the precision of estimates with a sample size of 150.^50^^,^^53^ As there may be differences in preferences by sex, the sample size was doubled to 300 to allow for stratification by sex assigned at birth.

(1) $$n\geq \frac{500\;*c\;}{t\;*a}c\;\text{is the maximum number of levels per}\text{attribute;}t\;\text{is the number of choice sets;}\text{and}\;a\;\text{is the number alternatives in each}\text{choice set}$$

### Screening and Recruitment

Participants for the ranking survey and DCE were recruited through Prolific. Eligible participants were adults (≥18 years old), residing in South Africa, enrolled at a tertiary-level institution, fluent in English, with a Prolific approval rating of ≥80% (eg, a score that indicates a participant’s reliability and quality as a study participant). Only eligible participants were offered an opportunity to complete the survey. A description of the study was provided, which included the expected completion time, the number of participants, how it could be completed (ie, tablet, computer, phone, etc), and reimbursement. Participation was voluntary, but those who elected to participate were redirected to Qualtrics to complete informed consent and the study survey. Participants who completed the ranking survey were not eligible to participate in the DCE.

### Piloting of Survey Instrument

We piloted the study among 10 participants to ensure the following: (1) the DCE could be completed within 30 minutes, (2) there were no issues accessing the Qualtrics survey and receiving payment upon completion, and (3) that there were no issues with study procedures such as the instructional video not playing nor with the wording of the DCE. We reviewed all 10 responses and found no issues with the tool or data (eg, questions being consistently skipped or attributes with blank values). Additionally, participants were able to contact A.Z./L.L. via a live chat through the Prolific platform during and after the survey if they experienced any issues. No participants made contact at any time. As there were no issues identified or changes made, the pilot sample was included in the analytic sample. The survey was conducted from June 26, 2024 to August 31, 2024.

### Ethics, Informed Consent, and Reimbursement

The study protocol was reviewed and approved by the Human Research Ethics Committee of the University of Witwatersrand (231115) and the Boston University Medical Center Institutional Review Board (H-44483). Both approved a waiver of written informed consent so that the participants could indicate consent online through an informed consent document shared with them on Qualtrics. The use of Prolific meant that all participants were anonymous to the research team, and no identifying information was provided. Participants were reimbursed for their participation at a rate of $12.00/hour (∼South African ZAR200/hour) as recommended by Prolific.^54^

### Statistical Analyses

Given that this was a forced choice, unlabeled DCE, a conditional logit model with dummy coding to analyze the choice data was conducted excluding the repeat choice set.^55^, ^56^, ^57^ Analyses were stratified by sex and socioeconomic status. A rudimentary measure of socioeconomic status level was used based on 3 standard questions regarding vehicle ownership, employment of household help, and medical insurance coverage. Participants who responded “No” to all 3 questions were defined as low, to 2 questions as medium, and to 1 or none as high socioeconomic status. We also conducted a conditional logit model that included an interaction between confidentiality and security level as a client’s preference for the length of personal data storage (confidentiality) might be influenced by how secure they considered the tool (security level). We conducted the following sensitivity analyses: (1) excluded those who completed the DCE in <10 minutes as the survey was intended to take 20-30 minutes to complete, (2) excluded those who were the fastest 10% of all participants, (3) excluded those who did not pass the internal consistency check (eg, the choice they chose for the duplicate choice set did not match the original choice set), (4) inclusion of the repeat choice set, and (5) conducted a mixed logit model analysis to evaluate the impact of preference heterogeneity which the conditional logit model does not necessarily account for. The DIRECT Checklist is provided in Supplemental Appendix 2 (available online at https://www.mcpdigitalhealth.org/).

## Results

### DCE Survey Participant Characteristics

Table 2 presents the baseline demographic characteristics of the 300 participants. All participants who started the discrete choice experiment completed all research activities. The mean age was 26.5 years (SD, 6.0), and most participants were Black, born in South Africa, heterosexual, and currently working for a wage. The survey took a median of 18.0 minutes (interquartile range [IQR], 14.7-21.3) to complete. Among the fastest 10% of participants, the survey took a median of 6.6 minutes (IQR, 5.8-8.5). Among the participants who completed the survey in <10 minutes, the survey took a median of 7.4 minutes (IQR, 5.9-9.2). Internal consistency was 71.8% (n=215) choosing the same choice in the repeat choice set as they did in the original choice set.

**Table 2:** Baseline Demographics Stratified by Sex and Overall (N=300)

	Male (n=150)	Female (n=150)	Total (N=300)
Age (y)	26.5 (5.3)	26.6 (6.7)	26.5 (6.0)
Ethnicity			
Black	130 (86.7)	127 (84.7)	257 (85.7)
Mixed	6 (4.0)	11 (7.3)	17 (5.7)
White	8 (5.3)	6 (4.0)	14 (4.7)
Asian	1 (0.7)	2 (1.3)	3 (1.0)
Other	4 (2.7)	3 (2.0)	7 (2.3)
Decline to answer	1 (0.7)	1 (0.7)	2 (0.7)
Nationality			
South African	137 (91.3)	141 (94.0)	278 (92.7)
Other	13 (8.7)	9 (6.0)	22 (7.3)
Country of birth			
South Africa	137 (91.3)	140 (93.3)	277 (92.3)
Other	13 (8.7)	10 (6.7)	23 (7.7)
Province			
Eastern Cape	9 (6.0)	12 (8.0)	21 (7.0)
Free State	5 (3.3)	8 (5.3)	13 (4.3)
Gauteng	83 (55.3)	82 (54.7)	165 (55.0)
KwaZulu-Natal	16 (10.7)	14 (9.3)	30 (10.0)
Limpopo	9 (6.0)	5 (3.3)	14 (4.7)
Mpumalanga	3 (2.0)	5 (3.3)	8 (2.7)
North West	8 (5.3)	9 (6.0)	17 (5.7)
Northern Cape	2 (1.3)	0 (0.0)	2 (0.7)
Western Cape	15 (10.0)	15 (10.0)	30 (10.0)
Institution type			
University	128 (85.3)	127 (84.7)	255 (85.0)
T-VET College	8 (5.3)	8 (5.3)	16 (5.3)
Other tertiary institution	14 (9.3)	15 (10.0)	29 (9.7)
Year at tertiary institution			
First	25 (16.7)	22 (14.7)	47 (15.7)
Second	33 (22.0)	25 (16.7)	58 (19.3)
Third	37 (24.7)	47 (31.3)	84 (28.0)
Fourth	36 (24.0)	39 (26.0)	75 (25.0)
Greater than fourth	19 (12.7)	17 (11.3)	36 (12.0)
Gender			
Female	0 (0.0)	149 (99.3)	149 (49.7)
Male	149 (99.3)	0 (0.0)	149 (49.7)
Transgender	1 (0.7)	1 (0.7)	2 (0.6)
Sexual orientation			
Heterosexual	144 (96.0)	126 (84.0)	270 (90.0)
Bisexual	2 (1.3)	17 (11.3)	19 (6.3)
Gay	3 (2.0)	0 (0.0)	3 (1.0)
Lesbian	0 (0.0)	5 (3.3)	5 (1.7)
Other	1 (0.7)	2 (1.4)	3 (1.0)
Socioeconomic status^a^			
Low	54 (36.0)	44 (29.3)	98 (32.7)
Medium	46 (30.7)	45 (30.0)	91 (30.3)
High	50 (33.3)	61 (40.7)	111 (37.0)
Currently working for a wage	89 (59.3)	107 (71.3)	196 (65.3)
Own a car	61 (40.7)	62 (41.3)	123 (41.0)
Employ domestic workers	45 (30.0)	58 (38.7)	103 (34.3)
Medical aid coverage			
Yes	55 (36.7)	69 (46.0)	124 (41.3)
No	90 (60.0)	77 (51.3)	167 (55.7)
Decline to answer	1 (0.7)	1 (0.7)	2 (0.7)
I do not know	4 (2.7)	3 (2.0)	7 (2.3)
Place last sought medical care			
Private setting^b^	74 (49.3)	90 (60.0)	164 (54.7)
Public setting^c^	72 (48.0)	52 (34.7)	124 (41.3)
Other^d^	3 (2.0)	4 (2.7)	7 (2.3)
Decline to answer	0 (0.0)	1 (0.7)	1 (0.3)
I do not know	1 (0.7)	3 (2.0)	4 (1.3)

a Based on the following questions: owning a car, employing a domestic work, and having medical aid coverage. Low: answer no to all 3 questions; medium: answer yes to only 1 of the 3 questions; and high: answer yes to at least 2 of the 3 questions.

b Private doctor, hospital, clinic, nurse, or chemist.

c Public health clinic or hospital.

d Includes traditional healer.

### Primary and Stratified Analyses

**Figure 2:**
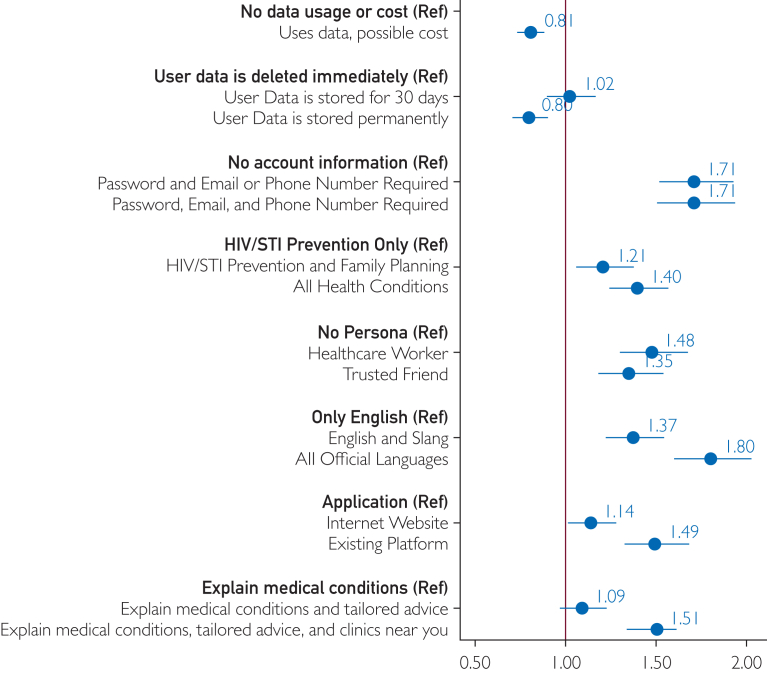
Primary findings from the discrete choice experiment using a conditional logit model with dummy coding (N=300).

Participants showed a strong preference for an AIPHA that securely stored their information by allowing them to create a password-protected account with personal identifiers included (odds ratio [OR], 1.71; 95% CI, 1.50-1.94; Figure 2; Supplemental Table 5, available online at https://www.mcpdigitalhealth.org/) compared with an AIPHA that included no account information or personal identification. They also had a strong preference for tools that allowed the use of any of the official South African languages (OR, 1.80; 95% CI, 1.60-2.02), or at least English in combination with local slang and vernacular (OR, 1.37; 95% CI, 1.22-1.54) compared with only being in English. Giving the AIPHA a personality of either a health care worker (OR, 1.48; 95% CI, 1.30-1.68) or a trusted friend (OR, 1.35; 95% CI, 1.18-1.54) was also preferred to having a chatbot agent with no personality. AIPHAs that could be used to discuss all health conditions (OR, 1.40; 95% CI, 1.24-1.57) or at least a combination of HIV/STI prevention information and family planning (OR, 1.21; 95% CI, 1.06-1.38) were preferred compared with an AIPHA only focused on HIV/STI prevention. AIPHAs that offered tailored advice and were able to direct the client to clinics near them were preferred to those tools that only explained the medical conditions more generically. It was also preferred if these tools could be accessed through existing platforms like WhatsApp (OR, 1.49; 95% CI, 1.32-1.68) or a website (OR, 1.14; 95% CI, 1.01-1.29) rather than through a bespoke application. Having a cost associated with using the AIPHA (OR, 0.81; 95% CI, 0.74-0.88) or an AIPHA that stored their questions and answers permanently (OR, 0.80; 95% CI, 0.70-0.90) were potential deterrents. There was a strong preference where if the AIPHA stored data at any level (either 30 days or permanently) there was also a strong preference requiring a password-protected account (30 days∗Password/Email OR, 2.00; 95% CI, 1.41-2.84; Permanently∗Password/Email/Phone OR, 1.73; 95% CI, 1.11-2.72) compared with an AIPHA that did not store the data and did not require a password-protected account. The odds ratio for the other interactions (30 days∗Password/Email/Phone OR, 1.26; 95% CI, 0.99-1.60; and Permanently∗Password/Email OR, 1.18; 95% CI, 0.98-1.42) did not show a strong effect. When stratified by sex, the results were similar with no notable differences (Supplemental Figure 1, available online at https://www.mcpdigitalhealth.org/; Swait and Louviere *P*=.19). When stratified by socioeconomic status, the results were similar for all characteristics except for language (Swait and Louviere *P*=.56). Participants in a low and medium socioeconomic status had a stronger preference for a tool that allowed the use of any of the official South African languages (low OR, 1.98; 95% CI, 1.61-2.44; medium OR, 1.90; 95% CI, 1.52-2.37) compared with participants of a high socioeconomic status (OR, 1.62; 95% CI, 1.33-1.97; Supplemental Figure 2, available online at https://www.mcpdigitalhealth.org/).

### Sensitivity Analyses

Sensitivity analyses 1-3 demonstrated that findings were consistent with the primary analyses (Supplemental Figures 3-5, available online at https://www.mcpdigitalhealth.org/), suggesting that neither the speed with which the survey was completed nor the fact that a participant had discordant results on the repeated choice set altered the overall results of the study. When using the repeat choice set instead (sensitivity analysis 4), the only notable change was participants had no preference for using a website over a bespoke application (OR, 1.00; 95% CI, 0.89-1.13; Supplemental Figure 6, available online at https://www.mcpdigitalhealth.org/). After accounting for preference heterogeneity, the beta coefficients from the mixed logit model were consistent with the beta coefficients from the conditional logit model (sensitivity analysis 5; Supplemental Table 6, available online at https://www.mcpdigitalhealth.org/).

## Discussion

Our study evaluated South African university students’ preferences for certain characteristics in an AIPHA. There is currently limited literature on the characteristics AIPHAs should include, given that only recent advancements in LLMs have allowed for the development of these assistants. Our results found that having an AIPHA that used local languages was password-protected, had secure account information, and provided personalized health advice including information on nearby clinics was most strongly preferred among South African university students. These characteristics align with preferences observed among individuals seeking in-person health care services in South Africa, reflecting consistent user priorities in both environments. 58, 59, 60

Participants’ strong preference to “converse” with AIPHAs in a local language, even when proficient in English, emphasizes the importance of language diversity in communicating health information and promoting equitable access to health care services.^61^ This is especially salient for LLM-powered tools, which historically have been developed in English and often perform poorly with African languages.^62^ AIPHAs may need the ability to code-switch within conversations. Ongoing efforts, such as those by Meta, aim to build AI models using African languages.^63^ A related concern is that much of the LLM training data come from high-income countries and have been found to perpetuate biases, especially against people from marginalized communities, people of lower socioeconomic status, non-Western identities, and people who experience greater difficulties in accessing care.^62^^,^^64^, ^65^, ^66^, ^67^ Therefore, any use of LLMs in South African health care must take this into account to avoid exacerbating health disparities and inequities.^68^, ^69^, ^70^, ^71^

Concerns around data security (eg, passwords and user accounts) and confidentiality (data retention and sharing) were characteristics that might influence South African tertiary students’ willingness to use AIPHAs. This was evident when evaluating the individual attributes and the interaction terms. Findings from the interaction terms indicate that if data are kept at any level, participants are also more likely to prefer some level of security to access the information, with a greater level of security preferred when data are stored permanently than when it is only stored for 30 days. This is particularly important in the South African context where HIV prevalence is high and discussing certain health topics with an AIPHA carries stigma and the potential risk of unintended disclosure. Although potential users of AI have expressed concerns regarding the security and privacy of AI tools, other research has also found that they may prefer AIPHAs to access stigmatized topics due to perceived confidentiality and a less judgmental space compared with communicating with an in-person health care provider.^26^, ^27^, ^28^, ^29^^,^^31^^,^^38^ Addressing security concerns and data privacy could enhance AIPHA acceptability and lead to appropriate and effective provision of health information.

As expected, characteristics that increased user burden were likely a deterrent to using an AIPHA (ie, requiring a specific application to be downloaded or incurring a cost such as the need to acquire data). This aligns with existing literature identifying cost and accessibility as barriers to accessing in-person health care services and predictive of successful uptake of health care interventions.^29^^,^^31^^,^^39^ In South Africa, mobile devices are often shared between friends and families so using existing applications with security features to ensure privacy, like WhatsApp, which has the ability to lock chats, may help ensure an individual’s information is kept private and prevent others from accessing their health-related chats.^72^ Finally, participants expressed a stronger preference for information regarding clinics near them and tailored medical advice. Prior studies have found that clinic proximity is an important factor as individuals are less likely to seek follow-up care if clinics are farther away.^73^, ^74^, ^75^ Furthermore, participants expressed a stronger preference for being able to access information on health conditions beyond HIV/STI prevention, suggesting that there is a desire for AIPHAs to serve as a “one-stop-shop” where participants can access information on multiple health-related topics. This supports evidence that patients desire integrated service delivery models when seeking health care.^73^, ^74^, ^75^, ^76^

Our analysis has several limitations. First, our study may not be representative of all young adult student populations as participants were recruited from Prolific. Although Prolific has been demonstrated to be appropriate for behavioral research, Prolific users self-select and may be of higher socioeconomic status with more exposure to technology than the general population. Furthermore, our mean age was 26 years, which is slightly higher than expected for undergraduate students. This is in part because over a third of our sample reported being in their fourth or higher year at a tertiary institution. Thus, findings from our study may not necessarily be generalizable to a younger university-age demographic who may be more familiar with AI. Second, we did not utilize any continuous attributes as there were no reliable user cost estimates for similar tools in this context; therefore, we are unable to comment on willingness to pay. Third, our study was conducted in English, and participants had to understand English to complete our study. Therefore, our results may not be generalizable to participants who are not fluent in English. Although the preference for multiple languages was strong, it is likely an underestimate and may have been stronger had there been greater language diversity in our sample. However, it is important to note that English is the language used for education in most South African tertiary institutions. Fourth, although our results, when stratified by socioeconomic status, remained consistent with our primary findings, these results may not be adequately powered, as we did not intentionally enroll equal numbers across each stratum, and hence, there were less than 100 participants in the low and medium socioeconomic status categories. Finally, our composite measure of socioeconomic status was based on standardized household questions and may not accurately reflect and capture all facets of socioeconomic status in South Africa. Specifically, they measured attributes that would generally be associated with a moderate-to-high level of socioeconomic status in this setting and could be indicative of where the student stayed (ie, with parents or student residence).

## Conclusion

In the evolving landscape of AI-driven health care, our work highlights the need to understand end-user preferences to ensure the full potential of this technology is realized. Our findings show South African tertiary students accessing health information value language accessibility and data privacy when engaging with an AIPHA while also desiring personality health advice and information on nearby clinics which requires data sharing. Future work should focus on evaluating whether these preferences vary for young adults outside of the tertiary education setting and if these preferences ultimately translate into uptake and improved health outcomes.

## Potential Competing Interests

Morris and Rech work for Audere that develops health applications powered by artificial intelligence.

## Ethics Statement

The study protocol was revised and approved by the University of Witwatersrand Human Research Ethics Committee (231115) and the Institutional Review Board of Boston University Medical Center (H-44483). A waiver of written informed consent was approved by both institutions so that participants could indicate consent online via an informed consent document shared with them on Qualtrics. The use of Prolific meant that all participants were anonymous to the research team and as such no identifying information was available.
