# Supplementary material for: Designing Artificial Intelligence-Powered Health Care Assistants to Reach Vulnerable Populations: A Discrete Choice Experiment Among South African University Students
Source: Mayo Clin Proc Digit Health. 2025 Jul 10;3(3):100248. doi: 10.1016/j.mcpdig.2025.100248 (PMC12337867; doi:10.1016/j.mcpdig.2025.100248)
Supplement: Supplementary Data [file mmc1.docx]

**Supplemental Tables and Figures Table of Contents**

**Supplemental Table 1.** Search terms for literature search conducted in PubMed from 2015-2023

**Supplemental Table 2.** The 13 characteristics and examples of each characteristic identified by the advisory team

**Supplemental Table 3.** Level Balance

**Supplemental Table 4.** Correlation matrix for orthogonality

**Supplemental Table 5.** Odds Ratios and 95% Confidence Intervals (CI) of Levels in the Primary Analysis

**Supplemental Table 6.** Beta Coefficients and Standard Deviations of Each Attribute from the Mixed Logit Analysis

**Supplemental Figure 1.** Results from the DCE stratified by A) Females and B) Males

**Supplemental Figure 2.** Results from the DCE stratified by A) Low Socioeconomic Status, B) Medium Socioeconomic Status, and C) High Socioeconomic Status

**Supplemental Figure 3.** Sensitivity Analysis 1: Excluding participants who completed the DCE in under ten minutes (N = 290)

**Supplemental Figure 4.** Sensitivity Analysis 2: Excluding the fastest 10% of participants (N = 287)

**Supplemental Figure 5.** Sensitivity Analysis 3: Excluding participants who did not pass internal consistency (N = 215)

**Supplemental Figure 6.** Sensitivity Analysis 4: Using the repeat choice set instead

**Supplemental Appendix 1.** Overview of the Rank Aggregation Analysis

**Supplemental Appendix 2.** Checklist for Reporting Discrete Choice Experiments

**Supplemental Table 1.** Search terms for literature search conducted in PubMed from 2015-2023

| **PubMed Search Terms** (2015-2023, English Language, excluded pre-print) | (“university student” or "university students" or “college student” or "college students” or "Universities"[Mesh] or “young adults” or “youth”) and (“barriers” or “facilitators” or "health services accessibility"[MeSH] or "Health Knowledge, Attitudes, Practice"[MeSH] or "Patient Acceptance of Health Care"[MeSH] or "acceptability" or "Patient Preference"[MeSH]) and ("mental health" or "HIV prevention" or "mental health services" or "HIV testing" or "Pre-Exposure Prophylaxis" or "PrEP" or "HIV/AIDS" or “primary care” or “ambulatory care” ) and "South Africa" |
| --- | --- |

**Supplemental Table 2.** The 13 characteristics and examples of each characteristic identified by the advisory team

| **Characteristic** | **Example** |
| --- | --- |
| *Confidentiality* | What happens to the data that you enter (Stored Forever, Deleted Immediately etc.)? |
| *Modality* | How do you access the tool (Application, Through existing application like WhatsApp, Website etc.)? |
| *Persona of the Tool* | Does the tool have a personality? (Health Care Worker, Family Member, etc.) |
| *Availability* | Can the tool be accessed at any time? (24/7, Clinic Hours) |
| *Ability to Communicate with a Real Person* | Can you talk to a real healthcare worker through the tool? (No, Get a call back, Direct Connection, etc.) |
| *Language* | Can you only speak English with the tool? (Yes, English but can understand local slang, Bilingual) |
| *Cost* | Does the tool require data to use or is it data free? |
| *Privacy/Security Level* | How secure is the tool (Nothing, Password, Requires email, etc.)? |
| *Provide Real Time Clinical Recommendations* | Does the tool offer specific medical advice to you or can it only give general information (Only gathers clinical information, Can explain medical conditions, Offers personal insights) |
| *Audio/Voice to Text* | Is there an option to interact with tool that does not require typing? (No, Audio and Voice to Text) |
| *Location Services* | Does the tool give you information about clinics specific to your location? (No, you can ask based on a specific location, Yes you have the option of it using your location)? |
| *Relationship* | Is the tool able to build a relationship over multiple sessions? (No, Yes - Can remember and remind about appointments or adherence) |
| *Services Provided* | What health topics can the tool talk about? (HIV/STI Only, HIV/STI/Family Planning, All Health) |

**Supplemental Table 3.** Level Balance

| **Attribute** | **Level** | **Appearances**  N (%) |
| --- | --- | --- |
| *Cost* | Free to use | 18 (50.0%) |
|  | Uses Data | 18 (50.0%) |
| *Confidentiality* | Data is deleted immediately and cannot be used in future uses | 13 (36.1) |
|  | Data is stored for 30 days and during the 30-day window data can be used anytime the electronic tool is accessed | 11 (30.6) |
|  | Data is stored permanently and can be used anytime the electronic tool is accessed | 12 (33.3) |
| *Security Level* | No information is required to use the tool | 11 (30.6) |
|  | A password and either a telephone number of email is required to use the tool | 13 (36.1) |
|  | A password, email, and telephone number is required to use the tool | 12 (33.3) |
| *Healthcare Topics the Tool Covers* | HIV/STI Prevention Information Only | 11 (30.6) |
|  | HIV/STI Prevention and Family Planning Information Only | 12 (33.3) |
|  | All health conditions including HIV/STI Prevention | 13 (36.1) |
| *Language* | Only English | 13 (36.1) |
|  | English and Slang (e.g., Language you would use with your friends) | 12 (33.3) |
|  | All official languages in South Africa | 11 (30.6) |
| *Personality of the Tool* | No Personality | 12 (33.3) |
|  | A trusted friend | 12 (33.3) |
|  | A health care worker | 12 (33.3) |
| *How You Will Access the Tool* | Application that needs to be downloaded | 13 (36.1) |
|  | Uses an existing application (Ex. WhatsApp) | 11 (30.6) |
|  | Internet website | 12 (33.3) |
| *Type of Advice the Tool Provides* | Explain medical conditions/procedures and likely course of treatment | 13 (36.1) |
|  | Explain medical conditions/procedures, likely course of treatment, and advice specific to you | 11 (30.6) |
|  | Explain medical conditions/procedures, likely course of treatment, advice specific to you, and recommend clinics near you | 12 (33.3) |

**Supplemental Table 4.** Correlation matrix for orthogonality

**Pairwise correlations**

| Variables | (1) | (2) | (3) | (4) | (5) | (6) | (7) | (8) | (9) | (10) | (11) | (12) | (13) | (14) | (15) | (16) | (17) | (18) | (19) | (20) | (21) | (22) | (23) |
| --- | --- | --- | --- | --- | --- | --- | --- | --- | --- | --- | --- | --- | --- | --- | --- | --- | --- | --- | --- | --- | --- | --- | --- |
| (1) cost_no | 1.000 |  |  |  |  |  |  |  |  |  |  |  |  |  |  |  |  |  |  |  |  |  |  |
| (2) cost_yes | -1.000* | 1.000 |  |  |  |  |  |  |  |  |  |  |  |  |  |  |  |  |  |  |  |  |  |
| (3) confid_relat_p~m | -0.058 | 0.058 | 1.000 |  |  |  |  |  |  |  |  |  |  |  |  |  |  |  |  |  |  |  |  |
| (4) confid_relat_no | -0.060 | 0.060 | -0.499* | 1.000 |  |  |  |  |  |  |  |  |  |  |  |  |  |  |  |  |  |  |  |
| (5) confid_relat_30 | 0.118 | -0.118 | -0.532* | -0.469* | 1.000 |  |  |  |  |  |  |  |  |  |  |  |  |  |  |  |  |  |  |
| (6) sec_lvl_no | 0.060 | -0.060 | 0.003 | 0.084 | -0.085 | 1.000 |  |  |  |  |  |  |  |  |  |  |  |  |  |  |  |  |  |
| (7) sec_lvl_email | 0.058 | -0.058 | 0.037 | -0.122 | 0.082 | -0.499* | 1.000 |  |  |  |  |  |  |  |  |  |  |  |  |  |  |  |  |
| (8) sec_lvl_both | -0.118 | 0.118 | -0.041 | 0.043 | 0.000 | -0.469* | -0.532* | 1.000 |  |  |  |  |  |  |  |  |  |  |  |  |  |  |  |
| (9) service_hiv | -0.060 | 0.060 | -0.122 | 0.084 | 0.043 | -0.047 | 0.129 | -0.085 | 1.000 |  |  |  |  |  |  |  |  |  |  |  |  |  |  |
| (10) service_hiv_fp | 0.118 | -0.118 | 0.082 | -0.085 | 0.000 | -0.085 | -0.041 | 0.125 | -0.469* | 1.000 |  |  |  |  |  |  |  |  |  |  |  |  |  |
| (11) service_all | 0.118 | -0.118 | 0.082 | -0.085 | 0.000 | -0.085 | -0.041 | 0.125 | -0.469* | 1.000* | 1.000 |  |  |  |  |  |  |  |  |  |  |  |  |
| (12) persona_hcw | 0.058 | -0.058 | -0.084 | -0.122 | 0.204 | 0.003 | 0.037 | -0.041 | 0.003 | -0.041 | -0.041 | 1.000 |  |  |  |  |  |  |  |  |  |  |  |
| (13) persona_friend | 0.118 | -0.118 | 0.082 | 0.171 | -0.250 | 0.043 | -0.041 | 0.000 | -0.085 | 0.125 | 0.125 | -0.532* | 1.000 |  |  |  |  |  |  |  |  |  |  |
| (14) persona_no | -0.181 | 0.181 | 0.003 | -0.047 | 0.043 | -0.047 | 0.003 | 0.043 | 0.084 | -0.085 | -0.085 | -0.499* | -0.469* | 1.000 |  |  |  |  |  |  |  |  |  |
| (15) lang_eng | 0.000 | 0.000 | 0.082 | -0.085 | 0.000 | 0.043 | -0.041 | 0.000 | 0.043 | 0.000 | 0.000 | -0.041 | -0.125 | 0.171 | 1.000 |  |  |  |  |  |  |  |  |
| (16) lang_eng_slang | 0.000 | 0.000 | -0.041 | -0.085 | 0.125 | 0.043 | 0.082 | -0.125 | 0.043 | -0.125 | -0.125 | 0.082 | -0.125 | 0.043 | -0.500* | 1.000 |  |  |  |  |  |  |  |
| (17) lang_multi | 0.000 | 0.000 | -0.041 | 0.171 | -0.125 | -0.085 | -0.041 | 0.125 | -0.085 | 0.125 | 0.125 | -0.041 | 0.250 | -0.213 | -0.500* | -0.500* | 1.000 |  |  |  |  |  |  |
| (18) mode_app | 0.058 | -0.058 | 0.037 | -0.122 | 0.082 | 0.003 | 0.037 | -0.041 | 0.003 | -0.041 | -0.041 | 0.037 | -0.041 | 0.003 | -0.041 | 0.082 | -0.041 | 1.000 |  |  |  |  |  |
| (19) mode_exist | -0.060 | 0.060 | 0.003 | -0.047 | 0.043 | 0.084 | 0.003 | -0.085 | -0.047 | 0.043 | 0.043 | 0.129 | -0.085 | -0.047 | 0.043 | 0.043 | -0.085 | -0.499* | 1.000 |  |  |  |  |
| (20) mode_inter | 0.000 | 0.000 | -0.041 | 0.171 | -0.125 | -0.085 | -0.041 | 0.125 | 0.043 | 0.000 | 0.000 | -0.164 | 0.125 | 0.043 | 0.000 | -0.125 | 0.125 | -0.532* | -0.469* | 1.000 |  |  |  |
| (21) rec_exp | -0.058 | 0.058 | 0.037 | 0.003 | -0.041 | 0.003 | 0.037 | -0.041 | 0.003 | -0.041 | -0.041 | 0.037 | -0.164 | 0.129 | -0.041 | 0.082 | -0.041 | 0.037 | 0.003 | -0.041 | 1.000 |  |  |
| (22) rec_spec | -0.060 | 0.060 | -0.122 | 0.215 | -0.085 | 0.084 | -0.122 | 0.043 | -0.047 | 0.043 | 0.043 | 0.003 | 0.043 | -0.047 | 0.043 | -0.085 | 0.043 | 0.003 | -0.047 | 0.043 | -0.499* | 1.000 |  |
| (23) rec_clin | 0.118 | -0.118 | 0.082 | -0.213 | 0.125 | -0.085 | 0.082 | 0.000 | 0.043 | 0.000 | 0.000 | -0.041 | 0.125 | -0.085 | 0.000 | 0.000 | 0.000 | -0.041 | 0.043 | 0.000 | -0.532* | -0.469* | 1.000 |
| **** p<0.01, ** p<0.05, * p<0.1* | | | | | | | | | | | | | | | | | | | | | | | |

**Supplemental Table 5.** Odds Ratios and 95% Confidence Intervals (CI) of Levels in the Primary Analysis

| **Attribute** | **Level** | **Odds Ratio**  **(95% CI)** |
| --- | --- | --- |
| *Cost* | Free to use | 1.00 *(Ref)* |
|  | Uses Data | 0.81 (0.74,0.88) |
| *Confidentiality* | Data is deleted immediately and cannot be used in future uses | 1.00 *(Ref)* |
|  | Data is stored for 30 days and during the 30-day window data can be used anytime the electronic tool is accessed | 1.02 (0.90,1.17) |
|  | Data is stored permanently and can be used anytime the electronic tool is accessed | 0.80 (0.70,0.90) |
| *Security Level* | No information is required to use the tool | 1.00 *(Ref)* |
|  | A password and either a telephone number of email is required to use the tool | 1.71 (1.51,1.93) |
|  | A password, email, and telephone number is required to use the tool | 1.71 (1.50,1.94) |
| *Healthcare Topics the Tool Covers* | HIV/STI Prevention Information Only | 1.00 *(Ref)* |
|  | HIV/STI Prevention and Family Planning Information Only | 1.21 (1.06,1.38) |
|  | All health conditions including HIV/STI Prevention | 1.40 (1.24,1.57) |
| *Language* | Only English | 1.00 *(Ref)* |
|  | English and Slang (e.g., Language you would use with your friends) | 1.37 (1.22,1.54) |
|  | All official languages in South Africa | 1.80 (1.60,2.02) |
| *Personality of the Tool* | No Personality | 1.00 *(Ref)* |
|  | A trusted friend | 1.35 (1.18,1.54) |
|  | A health care worker | 1.48 (1.30,1.68) |
| *How You Will Access the Tool* | Application that needs to be downloaded | 1.00 *(Ref)* |
|  | Uses an existing application (Ex. WhatsApp) | 1.49 (1.32,1.68) |
|  | Internet website | 1.14 (1.01, 1.29) |
| *Type of Advice the Tool Provides* | Explain medical conditions/procedures and likely course of treatment | 1.00 *(Ref)* |
|  | Explain medical conditions/procedures, likely course of treatment, and advice specific to you | 1.09 (0.97,1.23) |
|  | Explain medical conditions/procedures, likely course of treatment, advice specific to you, and recommend clinics near you | 1.51 (1.33,1.70) |

**Supplemental Table 6.** Beta Coefficients and Standard Deviations of Each Attribute from the Mixed Logit Analysis

| **Attribute** | **Level** | **Beta Coefficient**  **(Standard Deviation)** |
| --- | --- | --- |
| *Cost* | Free to use | *Ref* |
|  | Uses Data | -0.28 (0.06) |
| *Confidentiality* | Data is deleted immediately and cannot be used in future uses | *Ref* |
|  | Data is stored for 30 days and during the 30-day window data can be used anytime the electronic tool is accessed | 0.03 (0.08) |
|  | Data is stored permanently and can be used anytime the electronic tool is accessed | -0.27 (0.08) |
| *Security Level* | No information is required to use the tool | *Ref* |
|  | A password and either a telephone number of email is required to use the tool | 0.65 (0.08) |
|  | A password, email, and telephone number is required to use the tool | 0.66 (0.08) |
| *Healthcare Topics the Tool Covers* | HIV/STI Prevention Information Only | *Ref* |
|  | HIV/STI Prevention and Family Planning Information Only | 0.22 (0.08) |
|  | All health conditions including HIV/STI Prevention | 0.41 (0.07) |
| *Language* | Only English | *Ref* |
|  | English and Slang (e.g., Language you would use with your friends) | 0.38 (0.07) |
|  | All official languages in South Africa | 0.71 (0.08) |
| *Personality of the Tool* | No Personality | *Ref* |
|  | A trusted friend | 0.37 (0.09) |
|  | A health care worker | 0.48 (0.08) |
| *How You Will Access the Tool* | Application that needs to be downloaded | *Ref* |
|  | Uses an existing application (Ex. WhatsApp) | 0.48 (0.07) |
|  | Internet website | 0.17 (0.07) |
| *Type of Advice the Tool Provides* | Explain medical conditions/procedures and likely course of treatment | *Ref* |
|  | Explain medical conditions/procedures, likely course of treatment, and advice specific to you | 0.10 (0.07) |
|  | Explain medical conditions/procedures, likely course of treatment, advice specific to you, and recommend clinics near you | 0.48 (0.08) |

**Supplemental Figure 1.** Results from the DCE stratified by A) Females and B) Males


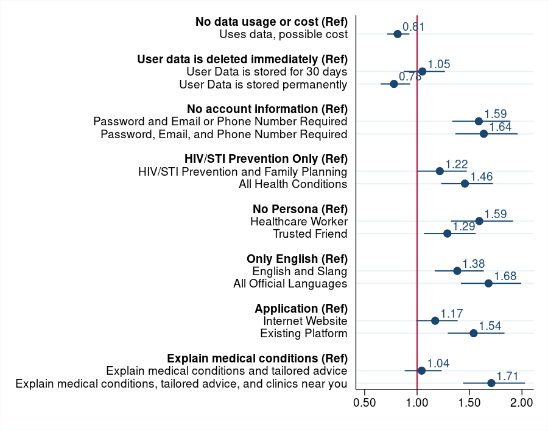


A)


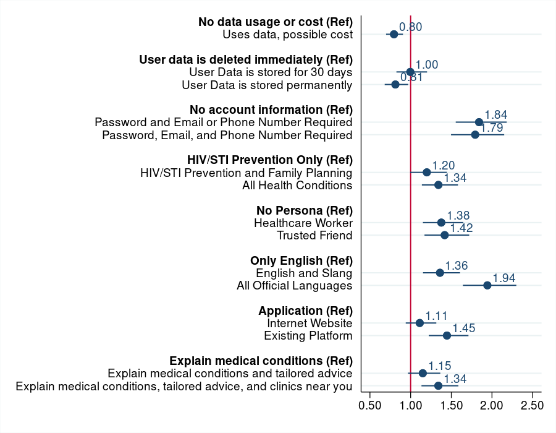


B)

**Supplemental Figure 2.** Results from the DCE stratified by A) Low Socioeconomic Status, B) Medium Socioeconomic Status, and C) High Socioeconomic Status


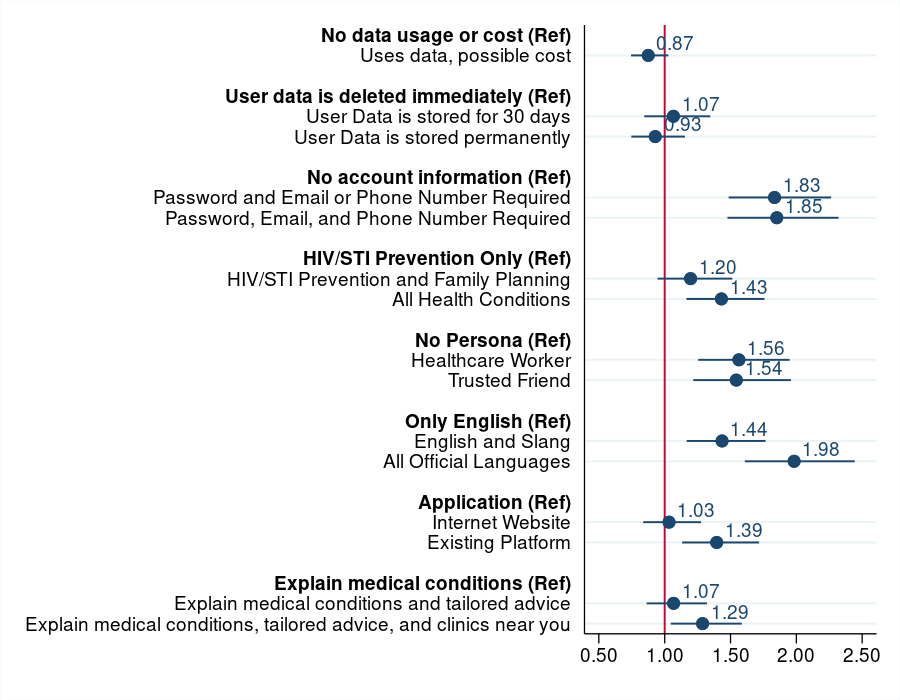

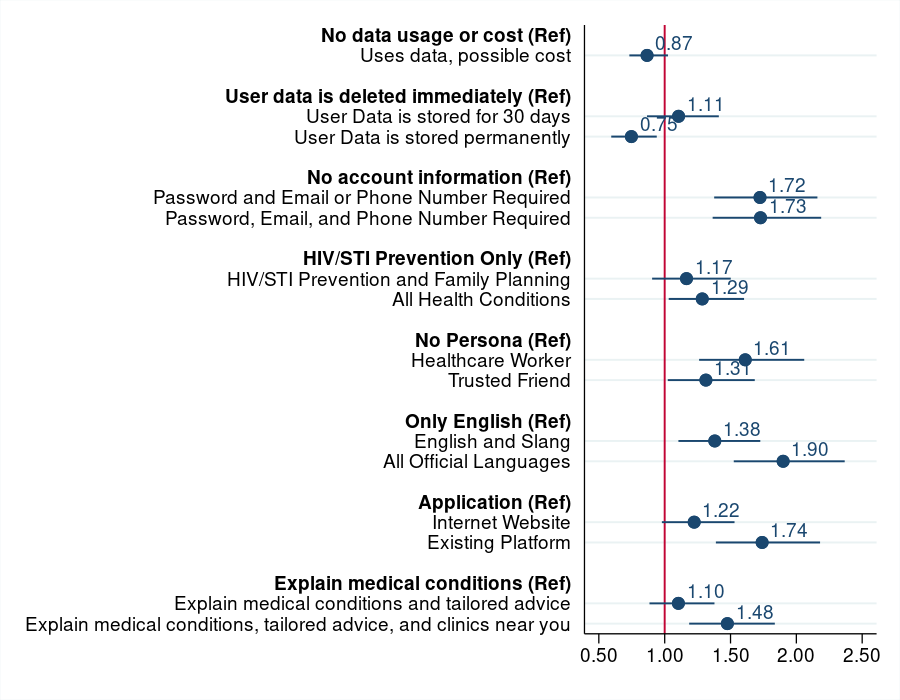

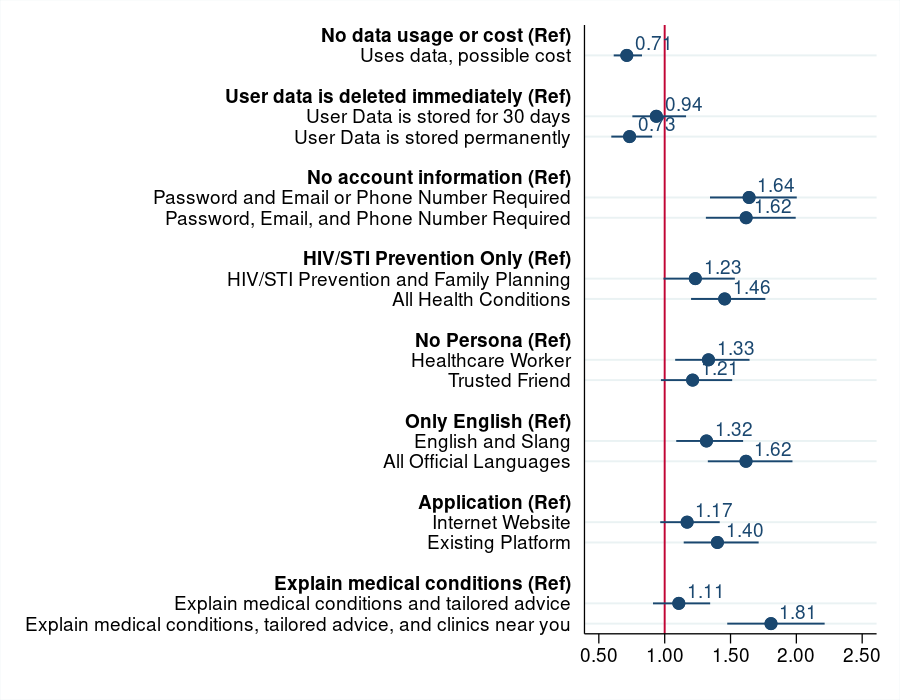


B)

C)

A)

**Supplemental Figure 3.** Sensitivity Analysis 1: Excluding participants who completed the DCE in under ten minutes (N = 290)


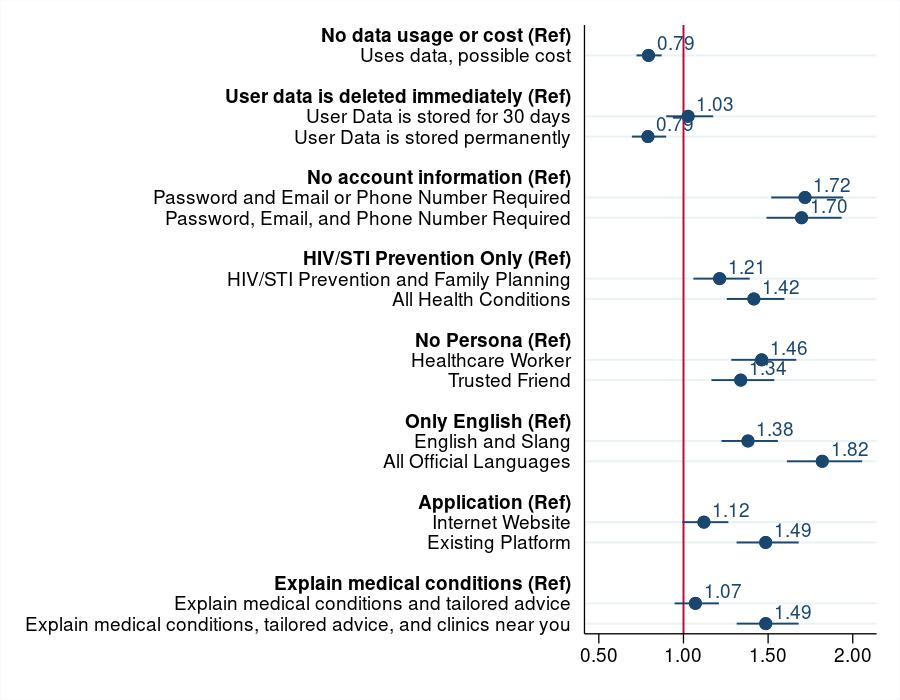


**Supplemental Figure 4.** Sensitivity Analysis 2: Excluding the fastest 10% of participants (N = 287)


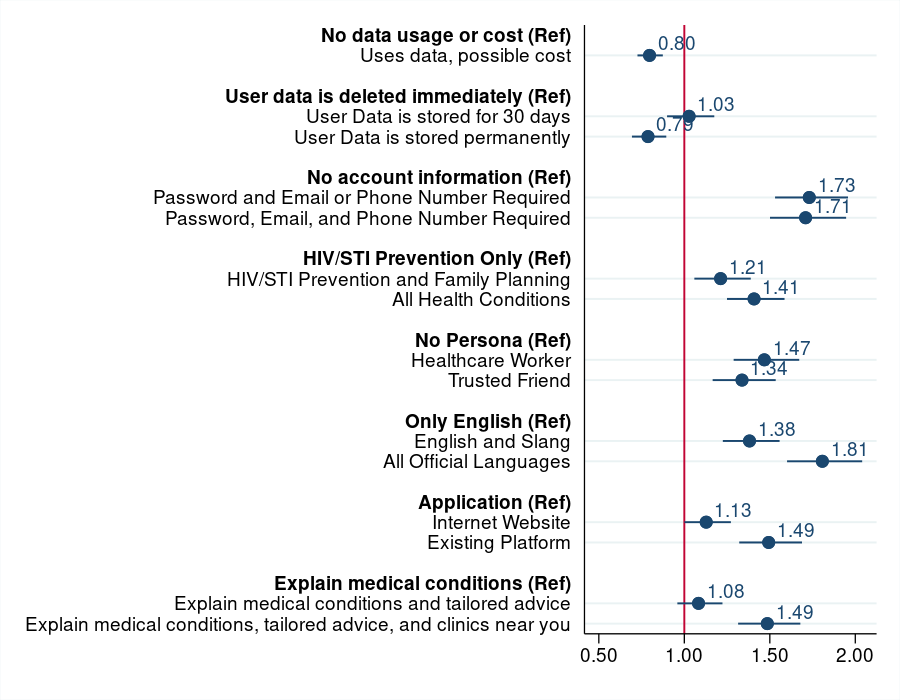


**Supplemental Figure 5.** Sensitivity Analysis 3: Excluding participants who did not pass internal consistency (N = 215)


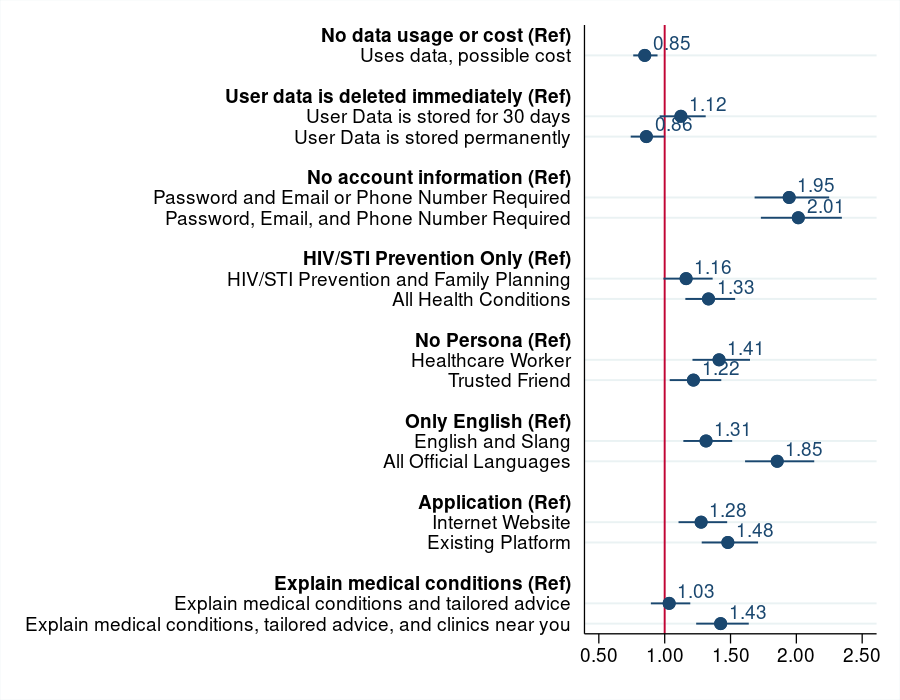


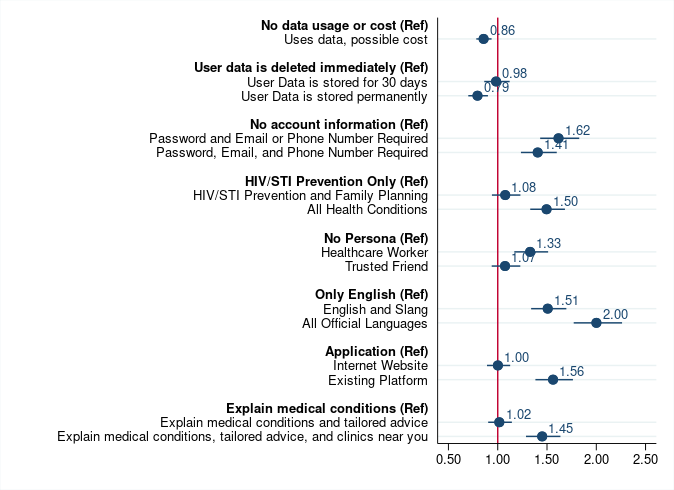
**Supplemental Figure 6.** Sensitivity Analysis 4: Using the repeat choice set instead

**Supplemental Appendix 1.** Overview of the Rank Aggregation Analysis

To create a single “super”-list that is reflective of all thirty responses, we conducted a rank aggregation using the Cross-Entropy Monte Carlo algorithm. At a high-level the algorithm uses a series of matrices to determine what the ideal list is. This involves four steps: 1) within each matrix, each cell has a uniform multinomial probability, 2) at each stage, a random sample of the matrices are generated via truncated multinomial sampling based on the cell probabilities of the select matrices. Third, based on the current sample for that stage and the list that is subsequently generated, the multinomial cell probabilities generated in Step 1 are updated with the goal of reducing the variability for the next random sample. Step 3 is repeated until convergence (Step 4) is achieved in which the list that is generated does not change for a given number of samples. The smaller the number of iterations and optimal value it takes to achieve convergence the better. It is important to note that if one were to run the algorithm several times, the “super”-list generated may vary slightly depending on the seed set and if the sample size is small.

**Supplemental Appendix 2.** Checklist for Reporting Discrete Choice Experiments

| **Section Item** | **Section/Paragraph Number** |
| --- | --- |
| *Purpose and Rationale* |  |
| 1. Describe the real-world context and decision maker that the hypothetical choice context seeks to replicate or inform | Introduction/2-3 |
| 1. Provide a rationale for using a DCE to answer the research question | Introduction/4 |
| *Attributes and Levels* |  |
| 1. Describe how attributes and levels were derived (e.g. literature review, interviews, focus groups, expert input) | Methods/1-2 |
| 1. Provide the final list of attributes and levels | Methods/2  Table 1 |
| *Experimental Design* |  |
| 1. Report the number of alternatives per choice set and whether they were labelled or unlabeled | Methods/3 |
| 1. Describe response options (e.g. forced choice, opt-out, status quo) | Methods/3 |
| 1. Describe the type of experimental design (e.g. orthogonal, D-efficient, Bayesian efficient, partial profile) | Methods/4 |
| 1. Describe which effects are identified in the design (e.g. main effects, higher order interactions, functional form) | Methods/9 |
| 1. Describe the number of choice sets, blocks and choice sets per block | Methods/3-4 |
| 1. Indicate how the experimental design was obtained (software, catalogue, other) | Methods/4 |
| *Survey Design* |  |
| 1. Provide a sample choice set and the instructions and background information given to respondents (e.g. providing the survey as an appendix) | Figure 1 |
| 1. Report any randomization (e.g. choice set order, attribute order, alternative order, framing effects) | Methods/4 |
| 1. Describe what was checked in piloting (e.g. understanding, respondent burden, timing, wording) | Methods/7 |
| 1. Report whether information from the pilot was used to update the experimental design (e.g. priors, functional form of attributes) or survey design | Methods/7 |
| *Sample and Data Collection* |  |
| 1. Report respondent inclusion/exclusion criteria | Methods/6 |
| 1. Describe how data were collected (e.g. mail, personal interview, web survey) | Methods/6 |
| 1. Report the response rate or cooperation rate, if possible | Not Applicable |
| 1. Report the final sample size and how the sample size was determined | Methods/5  Results/1 |
| 1. Describe respondent characteristics and representativeness of target population, if known | Not Applicable |
| *Econometric Analysis* |  |
| 1. Indicate coding of data (e.g. effects, dummy, continuous) including definitions | Methods/9 |
| 1. Report whether any respondents were removed and why (e.g. suspected fraudulent responses, rationality tests) | Methods/9 |
| 1. Provide the rationale for model choice (e.g. conditional logit, mixed logit, latent class) and assumptions (e.g. error variance) | Methods/9 |
| 1. Report model specification | Methods/9 |
| *Reporting of Results* |  |
| 1. Report the model performance, goodness of fit (if comparing models) | Not Applicable |
| 1. Describe methods used for analysis of model results (e.g. calculation of marginal rate of substitution, attribute relative importance, welfare gain) | Not Applicable |
| 1. Report measures of precision for the output(s) of interest (e.g. confidence intervals) and how these were derived | Results/2 |
